# NEIGHBORHOOD DISADVANTAGE AND THE ASSOCIATION OF PLASMA BIOMARKERS WITH COGNITION IN A COHORT OF DIVERSE ADULTS

**DOI:** 10.1093/geroni/igae098.1504

**Published:** 2024-12-31

**Authors:** Erica Fan, Sarah Royse, Alexandria Reese, Thomas Karikari, Tharick Pascoal, C Shaaban, Victor Villemagne, Ann Cohen

**Affiliations:** University of Pittsburgh School of Public Health, Pittsburgh, Pennsylvania, United States; University of Pittsburgh, Pittsburgh, Pennsylvania, United States; University of Pittsburgh School of Medicine, Pittsburgh, Pennsylvania, United States; University of Pittsburgh School of Medicine, Pittsburgh, Pennsylvania, United States; University of Pittsburgh, Pittsburgh, Pennsylvania, United States; University of Pittsburgh, Pittsburgh, Pennsylvania, United States; University of Pittsburgh School of Medicine, Pittsburgh, Pennsylvania, United States; University of Pittsburgh, Pittsburgh, Pennsylvania, United States

## Abstract

Studies of Alzheimer’s disease pathology have reported associations of plasma Aβ, tau, neurofilament light chain (NfL), and glial fibrillary acidic protein (GFAP) with cognition. However, it remains unclear how these biomarkers are associated with cognition in non-White populations affected by structural racism. Discriminatory policies have long shaped US neighborhood resources. Thus, our aim was to investigate whether neighborhood disadvantage modifies associations of plasma biomarkers with cognition in a diverse cohort of 136 adults ≥50 years. Predictors were plasma biomarkers (Aβ40, Aβ42, pTau217, pTau231, NfL, GFAP) measured using single molecule array or mass spectrometry. Outcomes included cognitive impairment and neuropsychological test scores. Our modifier, neighborhood disadvantage, was measured using area deprivation index (ADI), a composite of socioeconomic condition measures by census tract: higher ADI corresponds with worse conditions. In adjusted linear models, higher ADI, pTau217, pTau231, NfL, and GFAP, and lower Aβ40 and Aβ42 were separately associated with worse cognition. We found ADI*pTau217 interactions on immediate and delayed word recall. In those with low ADI, higher pTau217 was associated with worse immediate (β=-1.82, p=0.005) and delayed recall (β=-22.82, p<0.001); there were no such associations among high ADI participants. We also found an ADI*Aβ40 interaction on Rey figure immediate memory recall such that in low ADI participants, higher Aβ40 was associated with better recall scores (β=0.06, p=0.006), but this association was not significant in high ADI participants. Taken together, relationships between plasma biomarkers and cognition differ by neighborhood conditions, motivating further study of how neighborhood-level factors affect diagnosis and prognostication of cognition.

